# PMMA-Cement-PLIF Is Safe and Effective as a Single-Stage Posterior Procedure in Treating Pyogenic Erosive Lumbar Spondylodiscitis—A Single-Center Retrospective Study of 73 Cases

**DOI:** 10.3390/bioengineering9020073

**Published:** 2022-02-15

**Authors:** Moritz Caspar Deml, Emmanuelle N. Cattaneo, Sebastian Frederick Bigdon, Hans-Jörg Sebald, Sven Hoppe, Paul Heini, Lorin Michael Benneker, Christoph Emanuel Albers

**Affiliations:** 1 Department of Orthopaedic Surgery and Traumatology, Inselspital, University Hospital Bern, University Bern, 3010 Bern, Switzerland; emanuelecattaneo@bluewin.ch (E.N.C.); sebastian.bigdon@insel.ch (S.F.B.); sven.hoppe@insel.ch (S.H.); lorin.benneker@insel.ch (L.M.B.); christoph.albers@insel.ch (C.E.A.); 2 Department of Orthopaedic and Spine Surgery, Spital Thun, 3600 Thun, Switzerland; hans-joerg.sebald@spitalstsag.ch; 3 Department of Orthopaedic and Spine Surgery, Sonnenhof Spital, University Bern, 3006 Bern, Switzerland; paulheini@sonnenhof.ch

**Keywords:** spondylodiscitis, PMMA, polymethylmethacrylate, spinal implants, osteomyelitis, bony erosion, spinal infection, staphylococcus aureus, spine, discitis

## Abstract

Background: Surgical treatment for erosive pyogenic spondylodiscitis of the lumbar spine is challenging as, following debridement of the intervertebral and bony abscess, a large and irregular defect is created. Sufficient defect reconstruction with conventional implants using a posterior approach is often impossible. Therefore, we developed the “Cement-PLIF”, a single-stage posterior lumbar procedure, combining posterior lumbar interbody fusion (PLIF) with defect-filling using antibiotic-loaded polymethylmethacrylate (PMMA). This study first describes and evaluates the procedure’s efficacy, safety, and infection eradication rate. Radiological implant stability, bone-regeneration, sagittal profile reconstruction, procedure-related complications, and pre-existing comorbidities were further analyzed. Methods: A retrospective cohort study analyzing 73 consecutive patients with a minimum of a one-year follow-up from 2000–2017. Patient-reported pain levels and improvement in infectious serological parameters evaluated the clinical outcome. Sagittal profile reconstruction, anterior bone-regeneration, and posterior fusion were analyzed in a.p. and lateral radiographs. A Kaplan–Meier analysis was used to determine the impact of pre-existing comorbidities on mortality. Pre-existing comorbidities were quantified using the Charlson-Comorbidity Index (CCI). Results: Mean follow-up was 3.3 (range: 1–16; ±3.2) years. There was no evidence of infection persistence in all patients at the one-year follow-up. One patient underwent revision surgery for early local infection recurrence (1.4%). Five (6.9%) patients required an early secondary intervention at the same level due to minor complications. Radiological follow-up revealed implant stability in 70/73 (95.9%) cases. Successful sagittal reconstruction was demonstrated in all patients (*p* < 0.001). There was a significant correlation between Kaplan–Meier survival and the number of pre-existing comorbidities (24-months-survival: CCI ≤ 3: 100%; CCI ≥ 3: 84.6%; *p* = 0.005). Conclusions: The Cement-PLIF procedure for pyogenic erosive spondylodiscitis is an effective and safe treatment as evaluated by infection elimination, clinical outcome, restoration, and maintenance of stability and sagittal alignment.

## 1. Introduction

The pyogenic spondylodiscitis incidence ranges from between 0.4–2.4/100,000/year [1,2,3]. The lumbar spine is most commonly affected [4]. Conservative treatment with antibiotics for 6–12 weeks after isolating the causal microorganism is the first-line treatment [1,2,3,5,6,7]. Despite generally good results, conservative treatment may lead to persistent infection and segmental kyphosis with the risk of increasing instability, with reported failure rates of 12–17% [1,5]. Patients may suffer from persistent chronic back pain in up to 50%. They may require surgical intervention later on [1,3]. The main goal of surgery is to debride the intervertebral disc space and para-spinal infectious tissue or abscesses, decompression of the neural structures if necessary, and stabilize the affected segments [8,9]. However, an ongoing debate exists as to whether a supplementary anterior approach should be used in addition to a posterior approach [1,2,6]. Anterior lumbar procedures in this predominantly elderly patient population are potentially associated with increased procedure-related morbidity and complications. Of note, severe complications were reported in up to 15% of patients with prior abdominal operations [10,11]. Additionally, adequate filling of an osseous defect through a single posterior approach in patients with bony erosions of the adjacent endplates is technically demanding with conventional implants.

As a result of these considerations, we developed the “Cement-PLIF”, a single-stage posterior lumbar procedure to achieve sufficient debridement and defect-filling with antibiotic-loaded polymethylmethacrylate (PMMA), allowing for restoration of local anatomy, local antibiotic deposition, infection elimination, and maintenance of anterior column alignment.

This study was set up to evaluate the efficacy of this procedure as assessed by (1) infection eradication at minimum one-year follow-up; (2) radiological evaluation of segmental and implant stability, bone regeneration, posterior fusion, and sagittal profile reconstruction; (3) the incidence of procedure-related complications, and (4) comorbidity-related mortality.

## 2. Materials and Methods

**Figure 1:**
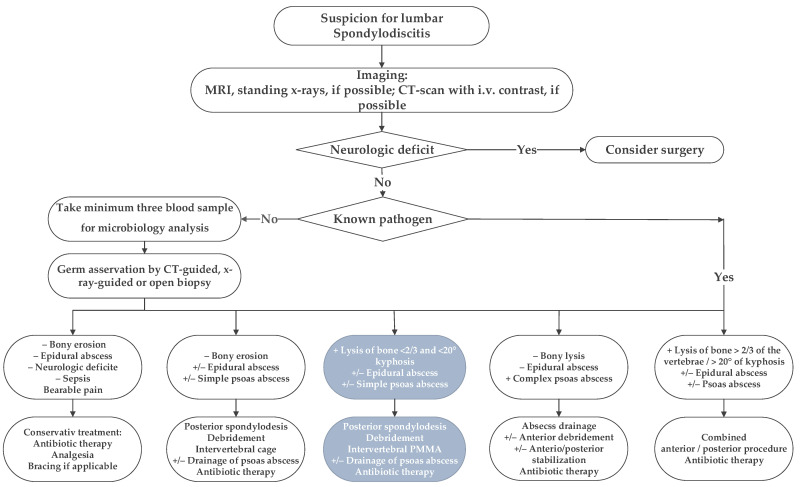
The in-house treatment algorithm for lumbar spondylodiscitis is illustrated. This study focused on the third group, marked with inverted colors, italic characters, and grey boxes.

**Figure 2:**
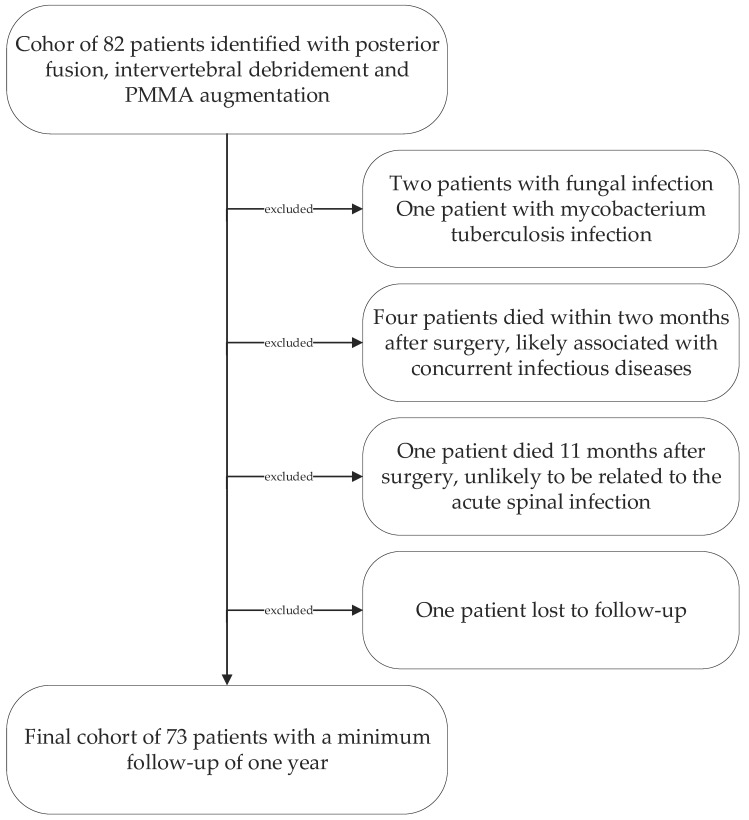
Patient cohort selection diagram.

Following institutional ethics board approval, we retrospectively reviewed all patients’ records treated surgically for lumbar pyogenic erosive spondylodiscitis between 2000 and 2017. According to our treatment algorithm for pyogenic spondylodiscitis (Figure 1), inclusion criteria for surgery were the failure of conservative treatment, defined by progressive segmental instability presenting increasing segmental kyphosis ≥5° combined with bony destruction, or not responding to antibiotic therapy. According to Boody and Saeed, further indications for surgery were an epidural abscess with neurological impairment, uncontrolled sepsis despite antibiotic treatment, progressive paraspinal abscess, or immobilizing pain [1,6]. Patients with the following conditions were excluded: non-pyogenic infections, under 18 years of age, active tumor disease, complete vertebra resections, concurrent multiple psoas abscesses not possible to treat by punctuation or conservatively, sacroiliac joint involvement, and those who did not consent for data analyses. Finally, 73 patients were included (Figure 2).

The diagnosis of erosive spondylodiscitis was based on clinical and laboratory findings (white blood count (WBC) and C-reactive protein (CRP)), radiological assessment (X-ray, CT, MRI, or scintigraphy) as well, as microbiological findings.

Clinical and radiological follow-up was performed at 2, 6, and 12 months postoperatively and annually after that. All patients underwent erect anteroposterior and lateral X-rays of the lumbar spine. MRI and CT imaging were only performed in a few cases (MRI 9/73; 12.3%; CT 11/73; 15.1%) due to new local symptoms.

WBC and CRP levels were monitored regularly throughout the hospital stay. Following discharge, the patients’ family doctors monitored them until normalization. Local and systemic signs of infection were documented. The pain was assessed using a Numeric Rating Scale (NRS) ranging from 0 (no pain) to 10 (maximal pain).

Segmental and overall lumbar lordosis (angle between the superior endplates of vertebra lumbar 1 and sacral 1) were assessed at any follow-up. Segmental lordotic angles were measured using the superior cranial and the inferior caudal endplates adjacent to the stabilized segment (Table 1). Since there is no classification for bony regeneration and fusion in spinal infections, we combined the classification by Lee with those described by Bridwell and Gruen to evaluate anterior bone regeneration and posterior fusion [12,13,14]. The grading is described in detail in Table 2. Two senior spine surgeons carried out the measurements. Blinding for the treatment was inherently not possible.

### 2.1. Demographics

The mean follow-up was 3.3 (range: 1–16; ±3.2) years. The mean age at surgery was 68.4 (range: 32–90; ±12) years; 28 patients were female (38.4%) (Table 1).

Before admission to our department, 29/73 patients (39.7%) had been hospitalized for an average of 11.4 (range: 2–26; ±9) days. Our mean department’s hospitalization time was 15.1 (range: 6–45; ±9.3) days. Patients were symptomatic for an average of 9.6 (range: 0.2–35; ±4.8) weeks before admission. Back pain was the most common clinical finding (*n* = 63/73; 86.3%). In 16/73 cases (21.9%), there was a concomitant epidural abscess. Eleven (11/73; 15.1%) patients presented with psoas abscesses; four were bilateral (36.4%). Eight (8/11; 72.7%) were drained by CT-guided biopsy prior to surgery. Three (3/11; 27.3%) were exhausted through the disc space intraoperatively. Neurological deficit was reported in 16/73 (21.9%) patients, ranging from mild to progressive nerve palsy. They fully recovered in 14/16 (87.5%) patients.

Blood cultures were drawn in 65/73 (89%) patients and were positive in 36/65 (55.4%). CT-guided biopsies were performed in 35/73 patients (47.9%) and were positive in 23/35 (65.7%). Intraoperative tissue samples were collected in all patients; 55/73 samples (75.3%) yielded positive. Overall, the causative organism was identified in 64/73 patients (87.7%). Polymicrobial infections were detected in three cases (3/73; 4.1%). The most frequently reported Gram-positive organisms were Staphylococcus species (26/67; 38.8%); the most found Gram-negative species were Escherichia coli and Pseudomonas aeruginosa (11/67; 16.7%) (Table 3). In 56 patients (56/73; 76.7%), the gentamicin covered the specific pathogen. In four patients (4/74; 5.5%), it was not tested, and in further four patients (4/74; 5.5%), it did not cover the pathogen. In one of the patients showing lysis Grade III around the cement, the gentamicin did not cover the pathogen found. Nevertheless, the systemic antibiotic therapy applied after surgery did cover the specific pathogen.

Following microbiological analyses and infectious disease recommendations, intravenous antibiotic therapy was administered for a mean of 15.4 days (range: 9–45; ±6.0). On average further oral antibiotic therapy was continued for another 10.6 weeks (range: 3–24; ±4.0).

In total, 88 lumbar segments were treated surgically. Sixty patients (60/73; 82.2%) received mono-segmental Cement-PLIF. In 11/73 (15.1%) cases, the infection extended to two segments; 2/73 (2.7%) patients suffered from three-segment involvement. The most frequently affected segment was L4/5 (25/88; 28.4%) (Table 4).

Comorbidities were evaluated using the Charlson Comorbidity Index (CCI) (Table 5) [15]. A Kaplan–Meier Survivorship analysis was performed with the endpoint defined as patient death 24 months postoperatively. For this analysis, all patients were allocated into two groups stratified according to a median-split of the CCI (median CCI: 3, group 1: *n* = 36, CCI ≤ 3; group 2: *n* = 37, CCI ≥ 3).

### 2.2. Surgical Procedure

**Figure 3:**
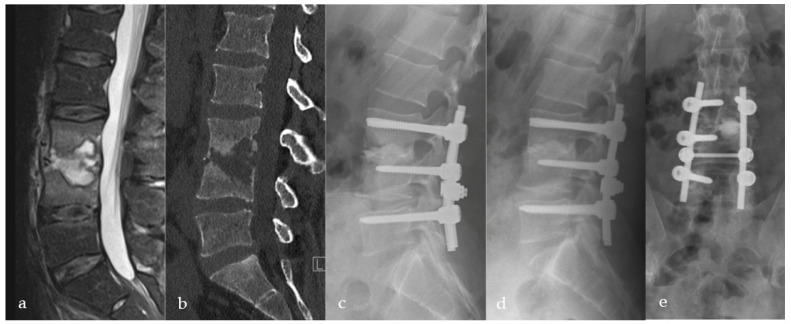
Thirty-eight-year-old male patient suffering from severe erosive spondylodiscitis with a ~40% bony defect in L3/4. Preoperative MRI and CT scans are shown in images (**a**,**b**). Immediately postoperatively (**c**) and one-year postoperatively (**d**) lateral and (**e**) a.p. X-rays show bony regeneration and clinical infection consolidation in the affected segment L3/4. L5 was included in the stabilization due to a weak bony situation during the operation to provide sufficient stability.

In all cases, an open pedicle screw fixation adjacent to the level of infection was performed. In 22/73 patients (30.1%), the high degree of osseous erosion did not allow for sufficient pedicle screw fixation in the affected segment only (>1/3 of vertebral body destruction or low bone quality). In these cases, pedicle screw fixation was extended to the vertebral body above or below the affected segments (Figure 3). The aim was to stabilize the minimal number of lumbar segments required to preserve motion. Subsequent decompression and a posterior inter-body fusion (PLIF) approach were carried out, and the intervertebral disc space was debrided. Microbiological samples were taken, followed by thorough irrigation. The cavity was filled with gentamicin-enriched PMMA bone cement (Optipac^®^Refobacin^®^Bone-Cement-R; 0.5 g Gentamycin/40 mL PMMA) through a cannula, monitored by bi-planar fluoroscopy. The vacuum-mixed cement usually takes about 8–10 min to harden; we start the application into the cavity about four minutes after mixing. To protect neural structures, we use a dissector or nerve retractor. Posterolateral bone grafting was subsequently performed in all cases using a local allograft (28/73; 38.4%), iliac-crest (18/73; 24.7%), and allograft (27/73; 37%). Patients were mobilized the same or the following day according to the extent of surgery, pain levels, and pre-operative mobilization status. All patients received instructions to avoid trunk rotation against the pelvis as well as extensive flexion/extension and side-flexion movements of the thoracic and lumbar spine. Neither a bandage nor a brace were used.

### 2.3. Statistics

Data were collected using Microsoft Excel Spreadsheet (Microsoft Office 2016, Microsoft Corporation, Redmond, WA, USA). Statistical analyses were performed with SPSS (Vers.25, IBM Corporation, Armonk, NY, USA). Kaplan–Meier Survivorship analysis was performed with the endpoint defined as patient death. The difference between the survivorship curves was calculated using the log-rank test. Normal distribution was tested using the Shapiro–Wilk test. Differences in clinical and radiological outcomes were computed using the Student’s *t*-test for normally distributed (Wilcoxon Signed-Rank Test for non-normally distributed) continuous data. Comparisons of independent data were calculated with the independent *t*-test for normally distributed data (Mann–Whitney U-Test for non-normally distributed data).

## 3. Results

Control of infection was achieved in all patients at the last follow-up. We noted significantly reduced mean CRP (pre-/postoperative: 112/41; *p* < 0.001) and WBC (pre-/postoperative: 11.7/8.3; *p* < 0.001) (Table 1). Pain decreased from a mean of 5.6 preoperatively to 2.2 at the last follow-up (Table 1).

**Figure 4:**
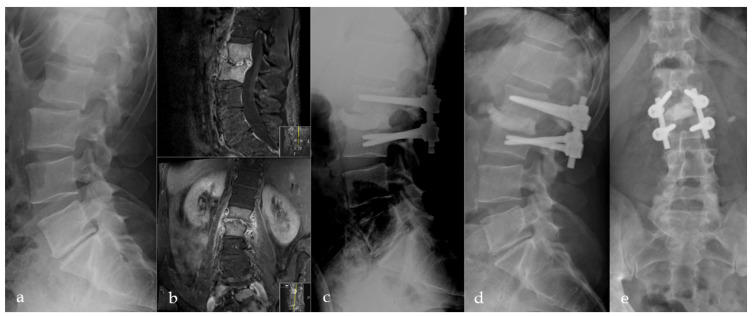
Initial X-ray shows a retrolisthesis L2/3 with mild segmental degeneration in a sixty-two-year-old male patient (**a**). Follow-up MRI due to persistent pain six weeks later shows pyogenic spondylodiscitis in L2/3 with progressive bony destruction (**b**). Directly postoperative (**c**) and 12 months (**d**,**e**) postoperative a.p. and lateral radiographs with anterior PMMA leakage, bone regeneration, and stable implants.

**Figure 5:**
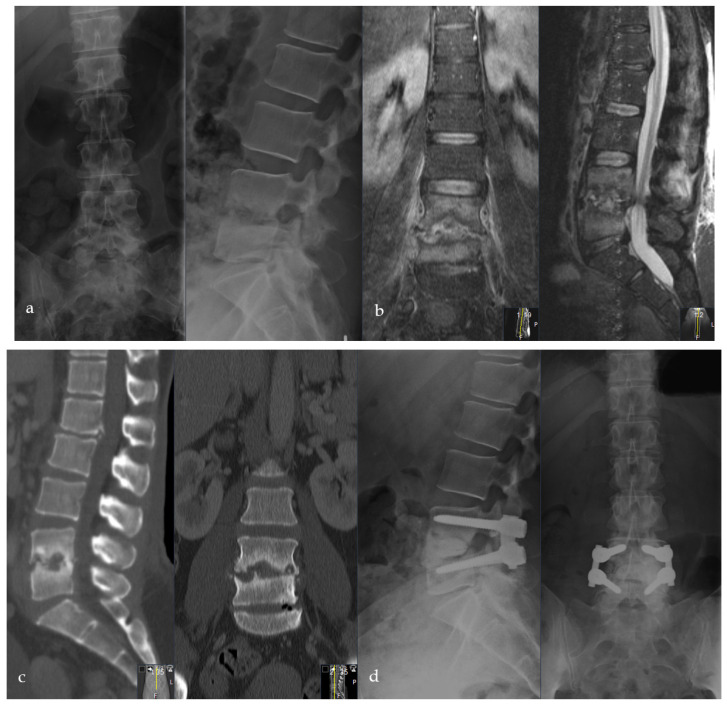

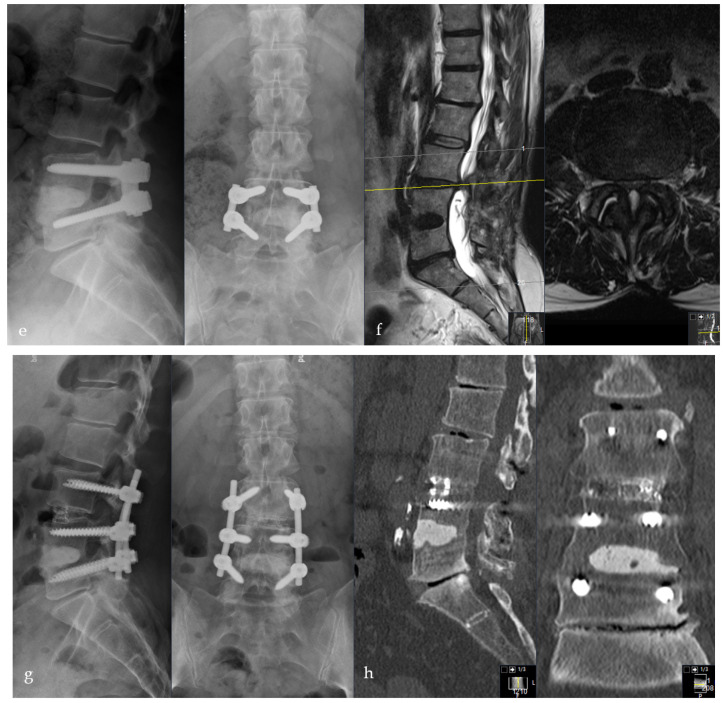
Female patient, 46-years old, pyogenic erosive spondylodiscitis L4/5 with staphylococcus aureus identified as the causative pathogen. Preoperative standing a.p. and lateral X-ray (**a**), MRI of the lumbar spine (**b**), and CT scan (**c**). Postoperative standing a.p. and lateral X-ray after Cement-PLIF L4/5 and postero-lateral fusion (**d**). Six-year follow-up with standing X-rays a.p. and lateral of the lumbar spine (**e**). Cranial adjacent segment disease (L3/4) is demonstrated by MRI (**f**). Adjacent segment fixation L3/4 after preoperative exclusion of reinfection by open biopsy with postoperative standing X-rays of the lumbar spine six years post-operatively (**g**). Sixteen-year follow-up CT scan of the lumbar spine with intact Cement-PLIF, anterior and posterior fusion, and infection elimination (**h**).

**Figure 6:**
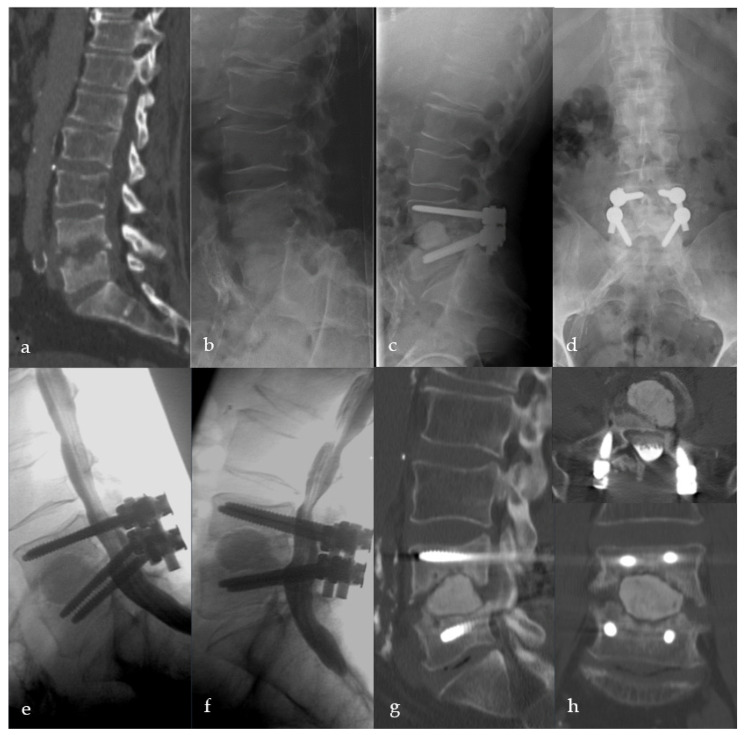
Sixty-two-year-old male patient with severe destructive spondylodiscitis L4/5. Preoperative CT-Scheme and lateral X-ray (**a**,**b**). Three-month follow-up radiograph (**c**,**d**). X-ray and CT scan one year after surgery showing anterior, intervertebral pseudarthrosis with stable posterior implants (**e**–**h**).

Radiological evaluation revealed osseous consolidation with implant stability (Grade-I and II) in 70/73 (95.9%) patients (Figure 3, Figure 4 and Figure 5). Three patients (3/73; 4.1%) showed inadequate or no fusion (Grade-III and IV). Two of them had lysis of >3 mm around the PMMA with stable posterior implants (Figure 6). The third patient presented with lysis >3 mm and pedicle screw breakage (Grade-IV). All three patients refused revision surgery. Inflammatory markers remained normal, and no clinical signs suggestive of infection were reported. We did not see further implant breakage or loosening.

Sagittal profile reconstruction was satisfactory at the most recent follow-up (mean lumbar lordosis pre-/postoperatively: 40/49; *p* < 0.001). Mean segmental lordosis also improved significantly (pre-/postoperatively: 5.6/14.3; *p* < 0.001) (Table 1).

Early secondary revision due to local postoperative complications was necessary for 5/73 (6.9%) patients. Two (2/73; 2.7%) patients had hematomas or psoas abscess progression, and one patient required a dural tear revision (1/73; 1.4%). Six months postoperatively, one patient showed local infection recurrence (1/73; 1.4%). The infection spread to the upper adjacent segment. We extended the stabilization and augmented the segment with PMMA either. After this revision, no further reinfection was recorded. Three (3/73; 4.1%) patients had late reinfection (13, 17, 36 months postoperatively) in another segment with a different pathogen. Six patients (6/73; 8.2%) had to be treated for adjacent fractures and degeneration (Figure 6). None of the Cement-PLIFs had to be revised or replaced (Table 6).

We saw two patients (2/73; 2.7%) with an anterior leakage of PMMA in the retroperitoneal space without clinical relevance or further complication (Figure 3). We did not see a relevant epidural leakage, which caused neurological damage or required revision. Furthermore, we had four patients (4/73; 5.5%) with a dural tear during surgery that was primary sutured. In one case, a secondary revision was necessary due to persistent liquor fistula. Two patients (2/73; 2.7%) with multilevel surgery showed a high blood loss > 2 L; they received erythrocyte concentrate transfusion. No further intraoperative complications occurred.

**Figure 7:**
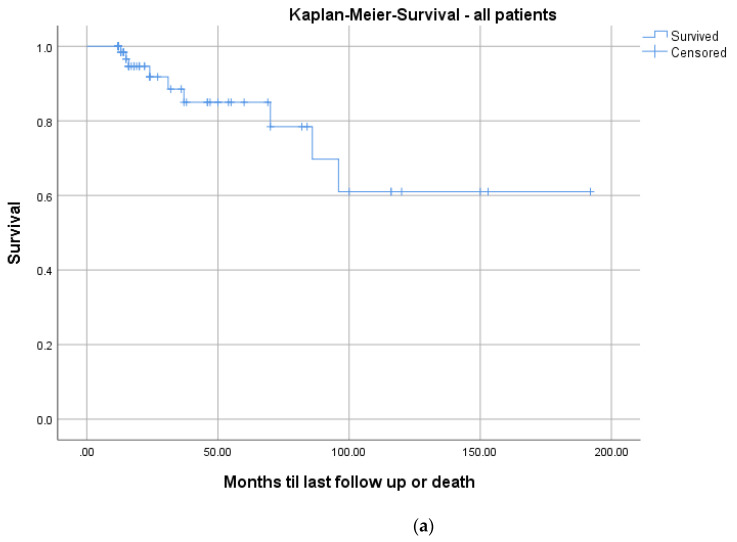

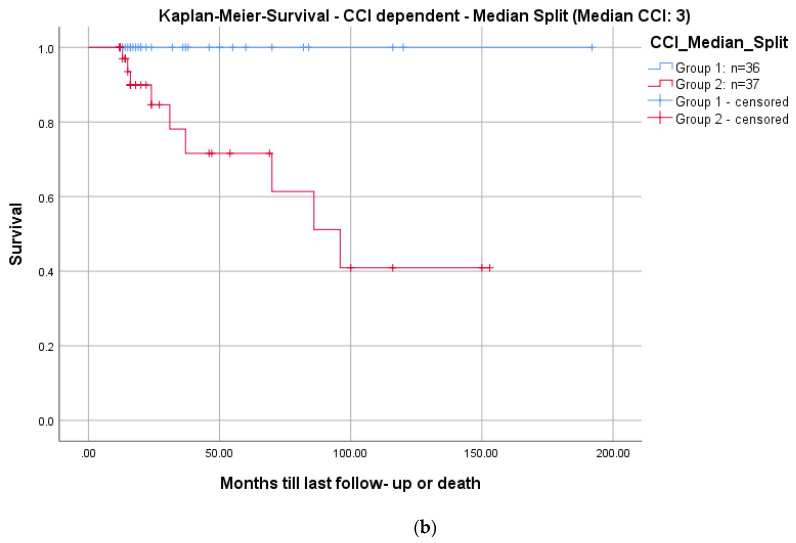
Kaplan–Meier survival curves for all patients (**a**) and differentiated by comorbidities determined with a median split of the CCI: CCI ≤ 3; *n* = 36 (blue graph) and >3; *n* = 37 (red graph), log-rank test: *p* = 0.005 (**b**).

The mean CCI for all patients was 3.3 (Table 4). In total, 9/73 patients (12.3%) died during the follow-up period after a mean of 3.6 years (range: 1.1–8.0; ±2.7). The mean CCI of these patients was significantly higher (mean: 7.3 ± 2.7; range: 5–13) than the remainder of the study group (mean: 2.8 ± 2.1; range: 0–11; *p* = 0.001). Kaplan–Meier survivorship analyses revealed prolonged survival in patients with a CCI ≤ 3 than patients with a CCI ≥ 3 (24 months follow-up: 100% vs. 84.6%; log-rank: *p* = 0.005; Figure 7).

## 4. Discussion

Pyogenic spondylodiscitis presents a significant health problem [1,2,3]. Even though first-line treatment includes obtaining a microbiological sample, intravenous and oral antibiotic therapy, analgesia, and optional bracing [1,2,3,5,6], ultimately, surgical treatment is considered in patients following failed conservative treatment [1,2,3,6,8,16]. However, there is ongoing debate regarding an adequate surgical treatment modality [2,3]. In our experience, spondylodiscitis without bony erosion of the adjacent vertebra can often be treated conservatively or with a conventional TLIF, PLIF, or XLIF-procedure, if necessary [17,18]. In erosive spondylodiscitis with large intervertebral osseous defects, anterior column reconstruction with standard intervertebral cages placed from the posterior may not sufficiently support the anterior column. There is also a dispute about the choice of implant for intervertebral stabilization [2]. In a study to evaluate the risk of foreign body implantation in infectious foci, Oga et al. documented a sufficient immune system response to prevent bacterial colonization of newly inserted foreign bodies [19]. Several studies have demonstrated that titanium is particularly suitable to improve stability and does not increase the risk of persistent infection [20]. PEEK implants show comparable results in spinal infectious diseases [17,18]. Long-term results for intervertebral PMMA-application compared to titanium and PEEK showed a higher rate of pseudarthrosis but no differences for the clinical outcome in cervical degenerative diseases [21,22]. Two studies document the use of standalone intervertebral PMMA in degenerative lumbar disc diseases in the elderly. They reported good clinical and radiologic results with the maintenance of coronal and sagittal profile reconstruction [23,24]. Thus, using PMMA for defect-filling and anterior column support seems advantageous for this subgroup of patients with large osseous defects (Figure 3 and Figure 6). In light of these facts, we developed the Cement-PLIF procedure in the 1990s, initially as a salvage procedure for patients with high-risk surgical profiles. PMMA is widely used in orthopedic surgical procedures, particularly to achieve adequate fixation in arthroplasties.

Rationales for using gentamycin-loaded cement are the Gram-positive spectrum of microorganisms and the good experiences with antibiotic-loaded PMMA in septic orthopedic procedures [1,25]. Other antibiotics, such as Vancomycin or Daptomycin, may also be used according to the resistances [25].

Only one case series of 14 patients reported on intervertebral PMMA application in cervical pyogenic spondylodiscitis with a mean follow-up of 3.7 years. Ozkan documented one surgical revision addressing reinfection. The PMMA was not augmented with antibiotics [26]. There is no literature on PMMA usage in lumbar or thoracic spondylodiscitis.

In our study, all cases showed total infection eradication at the last follow-up. One patient (1/73; 1.4%) presented with adjacent segment infection recurrence six months postoperatively. None of the Cement-PLIFs needed revision or had to be replaced.

Various studies assessed the outcomes in terms of infection eradication after the surgical treatment of mostly erosive spondylodiscitis [17,18,26,27,28,29,30]. Carragee evaluated 32 patients with a spinal infection. He reported one (4.5%) recurrence in 22 patients at the 10-year follow-up [27]. Bydon studied 118 patients undergoing surgical intervention in the cervical, thoracic and lumbar spine with and without stabilization. In the patients undergoing stabilization (*n* = 22), the authors noted 10% recurrence rates in the lumbar spine [28]. Lu reported on 36 patients treated with anterior corpectomy and expandable titanium cages with infection recurrence in two cases (5.6%) [29]. Vcelak retrospectively reviewed 31 patients with lumbar spondylodiscitis who received either posterior transmuscular stabilization with mini-open TLIF and defect-filling with autograft and tobramycin-loaded calcium-sulfate pellets (*n* = 23) and two-stage posterior stabilization with anterior titanium cage implantation (*n* = 8). The authors reported a 100% infection eradication rate for both groups [30]. Our technique seems to equal this with regard to infection eradication for erosive lumbar spondylodiscitis. However, it is essential to note that direct comparison of data in the literature is difficult due to the heterogeneity of surgical approaches and differing implants.

Bone regeneration around the PMMA combined with anterior or posterior fusion was seen in 70/73 (95.9%) patients (grade I and II). Bydon did not comment on fusion rates but reported three (3/22; 13.6%) revisions due to hardware failure [28]. Lu reconstructed the anterior bony defects with allograft or autograft combined with an expandable titanium cage through an anterior approach. There was also no comment on fusion rates in this study. However, the authors reported a complication rate of 0% regarding implant stability [29]. Vcelak documented 5/31 (16.1%) cases with posterior implant failure. All failures occurred in the mini-open TLIF group. Fusion rates were not reported [30]. However, the results presented by Vcelak highlight the importance of sufficient anterior stability with a failure rate of up to 16% [30]. The intervertebral filling of osseous defects with PMMA guarantees adequate anterior column support and superior bony regeneration comparable to the broader literature.

There is a well-documented correlation between correct sagittal profile reconstruction and postoperative quality of life [31]. We only have inconsistent data regarding the bone quality and osteoporosis in our cohort, often associated with a spinal infection. In our experience, local bone quality in erosive bony defects is virtually always compromised due to endplate destruction. However, the Cement-PLIF guaranteed mean segmental and global lumbar lordosis corrected by eight degrees (Table 1; Figure 3 and Figure 7). Many previous studies have not assessed lumbar sagittal parameters following surgical pyogenic spondylodiscitis procedures. Schomacher reported a significant cage-subsidence of at least 2mm in PEEK (67%) and titanium cages (75%). The segmental correction was significantly different from the most recent follow-up in both groups. They did not report on lumbar lordosis reconstruction [17]. Vcelak reported superior lumbar sagittal profile reconstruction when using anterior titanium cages compared to isolated posterior instrumentation [30]. Robinson and colleagues demonstrated significant sagittal profile reconstruction and maintenance after surgical treatment of 25 patients in thoracic und lumbar spondylodiscitis using expandable titanium cages at three years follow-up [32]. Despite limited comparability to previous studies, our procedure is not inferior to other techniques regarding sagittal profile reconstruction and maintenance over time.

Five patients (6.8%) had early procedure-related complications requiring revision surgery (Table 5). This rate is comparable to the complication rates reported before. Bydon noted a revision rate of 17.8%. One-half of the revisions were due to persistent infection [28]. Lu reported 8% with mild complications and 6% of 36 patients with severe complications [29]. Vcelak described necessary revisions in 10% of cases [30]. Open approaches cause extensive muscle injury leading to necrosis, providing a favorable environment for infection progression [10]. In our study, two hematomas and two cases of persistent infection required operative revisions. Our method could be used as the basis for a minimally invasive approach in the future, further decreasing these complications. This idea is reflected by Tschöke and Schomacher, who had zero revisions when using a minimally invasive TLIF approach with PEEK and titanium cages treating non-erosive spondylodiscitis [17,18].

In contrast to conventional implants, a potential side effect of PMMA is cement leakage. Significantly, there was no reported intravascular cement leakage. However, two cases (2/73; 2.7%) had anterior leakage, which was not clinically significant or needed revision surgery.

Clinical follow-up showed a significant decrease in pain and missing signs of infection in all cases at the last follow-up (Table 1). These results are comparable to data from the broader literature, albeit inhomogeneously presented [18,28,29,30]. We postulate that the significant improvement in pain is a combined result of the complete infection eradication and spinal stability with anterior column support, as seen in 95.9% of cases.

Mortality rates in pyogenic spondylodiscitis are up to 20% in registry data [33]. The Kaplan–Meier analysis, including all patients, demonstrated a survival rate of 91.8% at a 2-year follow-up (Figure 7a). These results are comparable to the literature [27,28,29,30,33]. Regarding the correlation of mortality to comorbidities, we saw significantly higher mortality in patients with a CCI ≥ 3 (*p* = 0.005; Figure 7b). There is no comparable analysis in the literature.

## 5. Limitations

Firstly, this is a retrospective case series without a control group. We cannot provide a control group because all patients with erosive pyogenic spondylodiscitis were treated using Cement-PLIF during the study period. Secondly, the gold standard to evaluate fusion is a CT scan. In our cohort, only 11/73 patients (15.1%) underwent a CT by the time of the most recent follow-up. However, the CT-scan results regarding fusion were consistent with those from X-ray analyses in all cases. Therefore, the use of X-rays in the follow-up to determine fusion should not alleviate the results significantly. Furthermore, we could not correlate the volume and the extent of defect filling by PMMA with radiologic results due to missing data. This will be addressed in future research projects. Lastly, due to the study’s retrospective nature, including patients up to 20 years ago, we cannot provide standardized patient-related outcome measures other than the NRS scale.

## 6. Conclusions

The Cement-PLIF procedure is a valuable option in treating erosive pyogenic spondylodiscitis of the lumbar spine with an infection elimination rate of up to 100% and bony regeneration of 96%. Segmental and global lumbar lordosis can be successfully restored and maintained. Complication rates are comparably low to those published in the literature and may be further reduced when modifying this technique to a minimally invasive procedure. Furthermore, mortality in lumbar pyogenic spondylodiscitis is dependent on pre-existing comorbidities.

## Figures and Tables

**Table 1:** Basic demographic data. (*), (**), (***), (****) and (*****) paired Student’s *t*-test *p* < 0.0001; ***NRS***: Number Rating Scale for subjective evaluation of pain level (0–10); Segmental lordosis: Angle between the upper and lower endplate of the adjacent vertebra to the infected disc space and endplates. Lumbar lordosis: Angle between the upper endplate of vertebra Lumbar 1 and Sacral 1.

Parameter	Values
Female (*n* [%])	28 (38.4%)
Average age (years)	68.1 (SD: 12.3; range: 32–90)
Debrided and augmented disc levels (*n*)	88
Stabilized segments (*n*)	121
Hospital stay (days)	15.1 (SD: 9.2; range: 6–45)
Previous lumbar spine operation (*n* [%])	14 (19.2%)
Mean operation time in minutes	166 (SD: 50: range: 60–420)
Average ASA-Classification (median [min–max])	3 (SD: 0.7; range: 1–4)
Mean blood loss (L)	0.71 (SD: 0.51; range: 0.2–2.5)
Mean CRP (mg/L, Normal < 5) at admission	112 (SD: 83; range: 9–322; CI: 91–133) *
Mean CRP (mg/L, Normal < 5) at discharge	41 (SD: 35; range: 2–144; CI: 33–50) *
Mean WBC (G/L, Normal range 3.0–10.5) at admission	11.7 (SD: 4.7; range: 3.6–22.9; CI: 10.5–12.8) **
Mean WBC (G/L, Normal range 3.0–10.5)at discharge	8.3 (SD: 2.6; range: 3.5–19.1; CI: 7.6–9) **
Mean NRS preoperatively	5.6 (SD: 2.9; range: 0–10; CI: 5.9–6.3) ***
Mean NRS at hospital discharge	4.8 (SD: 2.2; range: 2–10; CI: 4.3–5.4)
Mean NRS at last follow-up	2.2 (SD: 2.2; range: 0–10; CI: 1.7–2.8) ***
Preoperative segmental lordosis	5.6 (SD: 15.6; range: −29–43; CI: 1.7–9.5) ****
Postoperative segmental lordosis	14.3 (SD: 14.4; range: −19–49; CI: 10.7–17.9) ****
Preoperative lumbar lordosis	40.4 (SD: 15.5; range: −15–70; CI: 36.5–44.2) *****
Postoperative lumbar lordosis	48.8 (SD: 11.9; range: 12–72; CI: 45.8–51.8) *****

**Table 2:** New developed grading to evaluate bone regeneration, posterior fusion, and implant stability based on Lee et al., Bridwell et al. and Gruen et al. [12,13,14]. The halo sign is detectable, especially in anterior–posterior X-rays as lysis around a pedicle screw, suggestive of implant loosening.

	Grade I	Grade II	Grade III	Grade IV
Anterior Bone regeneration	+	+	-	-
Posterior Fusion mass	+	+	+	- +/− posterior implant failure
Lysis around the PMMA	none	<3 mm	≥3 mm	≥3 mm
Halo Ring Sign around pedicle screws (Lee et al.)	none	<1 mm	<1 mm	≥1 mm

**Table 3:** Microbiology results.

**Pathogen**	***n* (%)**
Evidence of causative pathogen	64/73 (87.7%)
Polymicrobial infections	3/73 (4.1%)
**Gram-positive bacteria**	**44/67 (65.7%)**
*Staphylococcus species*	*26/67 (38.8%)*
Staphylococcus aureus	15 (22.4%)
Staphylococcus aureus (methicillin-resistant)	1 (1.5%)
Coagulase-negative staphylococcus	9 (13.4%)
Staphylococcus lugdunensis	1 (1.5%)
*Streptococcus species*	*13/67 (19.4%)*
Beta-hemolytic streptococci	5 (7.5%)
Peptostreptococci	3 (4.5%)
Streptococcus viridans	2 (3.0%)
Streptococcus bovis	1 (1.5%)
Streptococcus pneumonia	1 (1.5%)
Streptococcus sanguinis	1 (1.5%)
*Others*	*5/67 (7.5%)*
Enterococcus faecalis	4 (6.0%)
Actinomyces	1 (1.5%)
**Gram-negative bacteria**	**23/67 (34.3%)**
Escherichia coli	7 (10.4%)
Pseudomonas aeruginosa	4 (6.0%)
Propionibacterium acnes	3 (4.5%)
Morganella morganii	2 (3.0%)
Yersinia	1 (1.5%)
Enterobacter aeruginosa	1 (1.5%)
Campylobacter fetus	1 (1.5%)
Klebsiella	1 (1.5%)
Arcanobacterium pyogenes	1 (1.5%)
Alcaligenes species	1 (1.5%)
Corynebacterium amycolatum	1 (1.5%)

**Table 4:** Affected lumbar segments.

Lumbar Level	*n* (%)
Th12/L1	6 (6.8%)
L1/2	7 (8.0%)
L2/3	21 (23.9%)
L3/4	18 (20.5%)
L4/5	25 (28.4%)
L5/S1	11 (12.5%)
Total	88
Ø/Patient	1.2

**Table 5:** Comorbidities classified according to the Charlson Comorbidity Index (CCI); (*) Student’s *t*-test: *p* = 0.001.

Comorbidity (Weight CCI)	*n*
Myocardial Infarction (1)	15
Congestive Heart Failure (1)	25
Vascular disease (1)	7
Peripheral Cerebrovascular disease (1)	9
Dementia (1)	6
Chronic Obstructive Pulmonary Disease (1)	5
Connective Tissue disease (1)	2
Peptic Ulcer disease (1)	4
Diabetes Mellitus uncomplicated (1)	18
Diabetes Mellitus with end-organ damage (2)	11
Moderate to severe Chronic Kidney Disease (2)	26
Hemiplegia (2)	4
Leukemia (2)	0
Malignant Lymphoma (2)	1
Solid Tumor (2)	11
Solid Tumor with metastatic diseases (6)	2
Liver disease mild (1)	11
Liver disease moderate to severe (3)	6
AIDS (6)	1
Mean CCI (all)	3.3 (SD 2.7; range: 0–13; CI: 2.7–4.0)
Mean CCI (died during follow–up; *n* = 9)	7.3 (SD: 2.6; range: 5–13; CI: 5.3–9.4) (*)
Mean CCI (survivors; *n* = 64)	2.8 (SD: 2.1; range: 0–11; CI: 2.3–3.3) (*)
Patients on hemodialysis	7 (9.6%)
Multi-substance abuse	27 (37%)

**Table 6:** Early and late local and non-local complications requiring revisions to the lumbar spine.

**Local Revisions**	***n* (%)**
*Early local revisions < 3 months after index surgery*	
Hematoma (posterior)	2 (2.7%)
Psoas abscess/hematoma	2 (2.7%)
Second-look	2 (2.7%)
Dural tear with fistula	1 (1.4%)
*Late local revisions ≥ 3 months after index surgery*	
Local infection in continuity with an adjacent segment with the same pathogen six months later	1 (1.4%)
**Non-Local Revisions**	***n* (%)**
Spondylodiscitis of another level due to different microorganisms (13, 17, and 36 months postoperatively)	3 (4.1%)
Adjacent fracture due to fall	3 (4.1%)
Adjacent segment degeneration	3 (4.1%)

## Data Availability

The datasets analyzed during the current study are available from the corresponding author on reasonable request.

## Abbreviations

WBC	White blood cell counting
CCI	Charlson Comorbidity Index
CRP	C-reactive protein
CT	Computed tomography
MRI	Magnetic resonance imaging
